# Red cell distribution width to albumin ratio is associated with asthma risk: a population-based study

**DOI:** 10.3389/fmed.2024.1493463

**Published:** 2024-12-11

**Authors:** Jinzhen Ding, Yixiang Zhang, Xiaoyang Chen

**Affiliations:** ^1^Department of Pulmonary and Critical Care Medicine, The Second Affiliated Hospital of Fujian Medical University, Quanzhou, Fujian, China; ^2^Respirology Medicine Centre of Fujian Province, The Second Affiliated Hospital of Fujian Medical University, Quanzhou, China

**Keywords:** red cell distribution width to albumin ratio, asthma, NHANES, risk, inflammation

## Abstract

**Background:**

The red cell distribution width to albumin ratio (RAR), a newly identified biomarker of inflammation, has been linked to a variety of inflammatory diseases. Asthma, a major burden on global health, is an inflammatory airway disease that is profoundly affected by inflammation. This study primarily sought to examine the influence of RAR on the risk of developing asthma.

**Methods:**

Data from 1999 to 2020 was gathered from the NHANES database. The impact of RAR on asthma risk and their non-linear relationship were clarified by multivariate logit and restricted cubic spline (RCS) analyses. Subgroup and interaction analyses collectively formed the sensitivity analysis for this study.

**Results:**

This study was based on an analysis of 54,161 individuals. RAR has been identified as an independent risk factor for asthma, according to logit analysis. The moderate and high RAR groups had a 16% [95% confidence interval (CI): 1.06–1.27] and 43% (95% CI: 1.30–1.58) higher risk, respectively, compared to the lowest group. Every 0.5 unit increase in RAR almost doubled the risk of asthma [odds ratio (OR): 1.82, 95% CI: 1.55–2.12]. There was no non-linear relationship between RAR and asthma risk, based on RCS analysis. Combining subgroup and interaction analyses results, all subgroups in this study showed consistent trends with the overall population.

**Conclusion:**

Notably, this article, the first to examine the relationship between RAR and asthma risk, unveiled a positive linear correlation between them. With an increase in RAR, whether analyzed as a categorical or continuous variable, asthma risk significantly increases. This finding was beneficial for clinicians to anticipate and assess the onset of asthma through stratified or dynamic management. Given RAR’s numerous advantages, its application in clinical settings held considerable promise.

## Introduction

Asthma, defined by restricted airflow, heightened airway reactivity, and airway structural changes, is a prevalent chronic inflammatory disease of the airways, (1) impacting diverse age groups globally. Analysis from the global burden of disease (GBD) database exhibited that in 2019, around 262 million individuals globally were affected, with an age-standardized rate of 3.4% (2). Asthma, a highly prevalent disease, also significantly contributed to premature death and decreased quality of life. Statistics revealed that in 2019, around 460,000 deaths from asthma occurred, with an age-standardized death rate of 5.8 per 100,000 people. Approximately 210,000 disability-adjusted life years (DALYs) were recorded, with an age-standardized rate of DALYs at about 273 per 100,000 (2).

Red cell distribution width (RDW) reflected the variability in red blood cell volume, a commonly used laboratory marker in clinical settings. RDW, typically used to differentiate types of anemia (3), has recently been shown to correlate with the prevalence and mortality of several inflammatory conditions, including chronic obstructive pulmonary disease (COPD) (4–6), cancer (7, 8), stroke (9, 10), sepsis (11), and diabetes (12, 13). Albumin, crucial for indicating nutritional status and inflammation, exhibited anti-inflammatory and antioxidant (14, 15). Relative to those with low albumin, individuals with high albumin exhibited significantly lower levels of pro-inflammatory cytokines like TNF and CRP (16). These findings suggested that RDW and albumin were associated with inflammation, oxidative stress, and nutrition, each reflecting these pathological aspects from distinct perspectives. Therefore, given their significant roles in physiological functions and disease assessment, integrating them into a composite biomarker has substantial value for predicting inflammation-related diseases.

The RDW to albumin ratio (RAR), derived from RDW/albumin, was linked to multiple inflammatory diseases, such as diabetes and related complications (17–19), rheumatic conditions (20), sepsis (21), stroke (22), and heart failure (23). Studies that have integrated both the NHANES and UK Biobank databases indicated that a higher baseline RAR was strongly linked to an increased risk of all-cause and cause-specific mortality in the general population (24). Asthma, an inflammatory airway disease, was widely recognized to be profoundly affected by inflammation. Therefore, a possible relationship between RAR and asthma might exist. Sadly, there were currently no studies examining the impact of RAR on asthma.

The core objective of this study was to explore the impact of RAR on asthma risk and to further analyze the non-linear relationship between them. Additionally, identifying the asthma population susceptible to changes in RAR facilitated early recognition of high-risk groups by healthcare professionals, enabling refined individualized management.

## Materials and methods

### Study population

**FIGURE 1:**
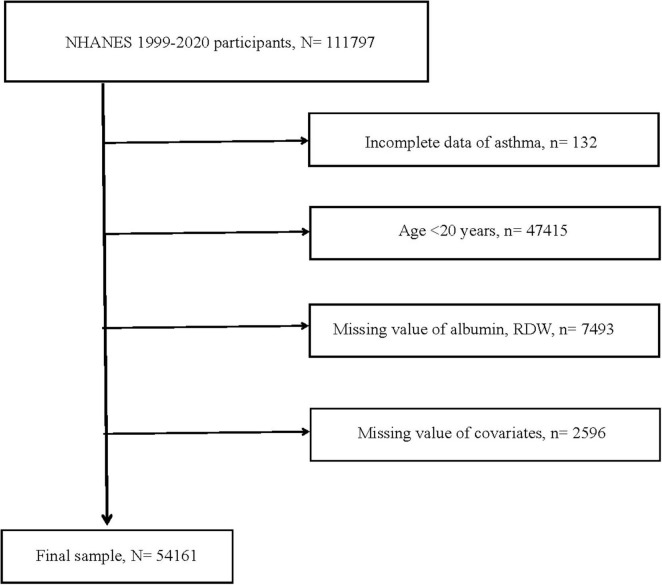
Flow chart of study participants. RDW, red cell distribution width; *N*, the number of patients being included; *n*, the number of patients being excluded.

This study’s data were obtained from NHANES, a nationally representative continuous large sample survey, which collected information on population nutrition, health, diseases, and risk factors through various methods including clinical measurements, laboratory tests, and questionnaires. Between 1999 and 2020, across eleven survey cycles, a total of 111,797 participants were included. Among them, 132 were excluded because of incomplete asthma data. Participants younger than 20 years old, totaling 47,415, were excluded. Subsequently, 7,493 cases were excluded due to missing RDW and albumin data. Due to incomplete information regarding demographic characteristics [age, gender, race, and body mass index (BMI)], medical history (smoking history, history of hypertension or diabetes), and laboratory tests (globulin and eosinophil percentage), a total of 2,596 were excluded. Ultimately, a total of 54,161 participants qualified for inclusion. Figure 1 illustrated the full inclusion and exclusion process for this study. In addition, education status, heart diseases, stroke, cancer, and other inflammation biomarkers (CRP and NLR) were collected from NHANES to included in further analyses to examine if the RAR captured the risks beyond these biomarkers. The detailed results were displayed in Supplementary Tables 1–3 and Supplementary Figure 1.

### Asthma

Asthma diagnosis relied on a standardized questionnaire. In particular, when participants answered “yes” to the question “Have you ever been told you have asthma?,” they were regarded as having asthma.

### RAR

RAR was formulated from RDW and albumin, represented as RAR = RDW/albumin. Participants were equally categorized into three groups based on RAR levels: low (2.08–2.93), moderate (2.93–3.27), and high (3.27–10.22).

### Covariates

**FIGURE 2:**
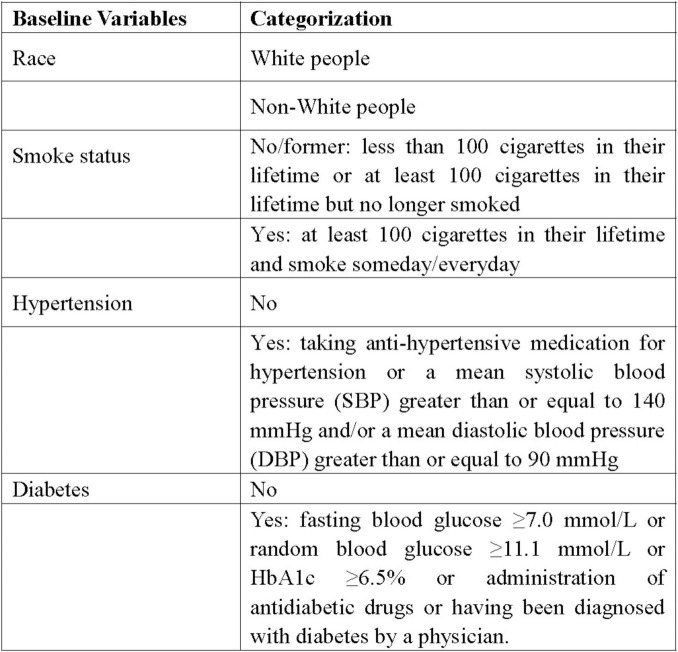
The categorization of baseline variables.

Data on demographic features (age, gender, race, and BMI), past conditions (history of smoking, hypertension, diabetes), and laboratory tests (globulin and eosinophil percentage) were extracted from the NHANES database. Racial categorization was routinely divided into White people and non-White people groups. Non-smokers and former smokers were merged into one group, with smokers forming the other group. According to history of hypertension and diabetes, individuals were split into yes and no groups. Figure 2 displayed the specific definitions and classification standards of the variables.

### Statistical analysis

Differences between baseline data were discerned using the *t*-test and chi-squared test, with the former for continuous variables and the latter for categorical variables. The influence of RAR on asthma risk was established through multivariate logit analysis. In the crude model, only the single RAR indicator was included. In model 1, demographic features (age, gender, race) were added as covariates in addition to RAR. Model 2 involved a comprehensive analysis of 10 variables, including RAR, age, gender, race, BMI, globulin, eosinophil percentage, history of diabetes, hypertension, and smoking. The results of this model were the most critical aspect of the study. Further result analysis relied mainly on the outcomes of model 2. Furthermore, non-linear relationship was a major focus of this research. Restricted cubic spline (RCS) analysis was used to ascertain the non-linear association between RAR and asthma risk. Should they exist, recursive algorithms were applied to identify inflection points, and segmented logit analysis was used to ascertain threshold effects. Moreover, an extra analysis was performed, where RAR was directly included as a continuous variable, assessing the dynamic influence of every 0.5 unit change on asthma risk. This study’s robustness was augmented by sensitivity analysis, including stratified and interaction analyses. For more in-depth analysis, BMI was split into groups of ≥ 25 and < 25. Globulin was divided into > 3 and ≤ 3 groups. The eosinophil percentage was categorized into groups of > 8 and ≤ 8. The stratified analysis results were used to identify high-risk groups. Interaction analysis was conducted to determine the presence of interactions between various covariates and RAR. *P*-values less than 0.05 were deemed statistically significant. All statistical analyses were performed using R version 4.3.1. The study was meticulously executed according to methods recommended by NHANES.

## Results

### Baseline characteristics

The analysis was conducted on a cohort of 54,161 individuals. participants were segmented into low, moderate, and high groups according to their RAR levels. The groups numbered 17,994, 18,028, and 18,139 individuals with mean (SD) RARs of 2.72 (0.00), 3.08 (0.00), and 3.66 (0.00), respectively. From Table 1, differences at baseline levels between the moderate and high groups and the low group could be observed. With the exception of smoking history, other variables demonstrated notable differences. Specifically, individuals in the moderate and high groups were older, with a higher proportion of females and Non-White people, greater obesity (higher BMI values), and higher rates of hypertension and diabetes, along with higher average concentrations of globulin and RDW, but lower average albumin levels. Further analysis revealed a similar trend, as detailed in Supplementary Table 1.

**TABLE 1:** Baseline characteristics of patients with asthma.

Characteristics	RAR				
	**Total**	**Low group**	**Moderate group**	**High group**	***P***
Participants, *n*	54,161	17,994	18,028	18,139	
RAR, mean	3.09 (3.08, 3.10)	2.72 (2.72, 2.73)	3.08 (3.08, 3.08)	3.66 (3.65, 3.67)	< 0.0001
Age, year	47.37 (47.04, 47.71)	42.18 (41.76, 42.61)	49.10 (48.63, 49.56)	53.05 (52.57, 53.52)	< 0.0001
Gender, *n* (%)					< 0.0001
Male	26,764 (48.82)	10,938 (59.41)	8,861 (47.30)	6,965 (34.76)	
Female	27,397 (51.18)	7,056 (40.59)	9,167 (52.70)	11,174 (65.24)	
Race, *n* (%)					< 0.0001
White people	23,489 (68.40)	9,167 (73.94)	7,914 (68.91)	6,408 (59.40)	
Non-White people	30,672 (31.60)	8,827 (26.06)	10,114 (31.09)	11,731 (40.60)	
BMI, Kg/m^2^	28.86 (28.74, 28.98)	26.82 (26.69, 26.94)	29.01 (28.85, 29.17)	31.75 (31.55, 31.96)	< 0.0001
Albumin, g/dL	4.27 (4.26, 4.28)	4.53 (4.52, 4.54)	4.23 (4.22, 4.23)	3.93 (3.92, 3.94)	< 0.0001
RDW, %	13.09 (13.06, 13.11)	12.32 (12.30, 12.34)	13.01 (13.00, 13.03)	14.34 (14.30, 14.38)	< 0.0001
Eosinophil percentage, %	2.81 (2.79, 2.84)	2.75 (2.72, 2.78)	2.86 (2.82, 2.90)	2.85 (2.81, 2.89)	< 0.0001
Globulin, g/dL	2.89 (2.88, 2.91)	2.79 (2.78, 2.81)	2.88 (2.86, 2.89)	3.06 (3.05, 3.08)	< 0.0001
Smoke status, *n* (%)					0.27
No/former	42,972 (78.95)	14,164 (78.46)	14,414 (79.14)	14,394 (79.46)	
Yes	11,189 (21.05)	3,830 (21.54)	3,614 (20.86)	3,745 (20.54)	
Hypertension, *n* (%)					< 0.0001
No	31,487 (63.46)	12,405 (72.01)	10,422 (61.94)	8,660 (52.45)	
Yes	22,674 (36.54)	5,589 (27.99)	7,606 (38.06)	9,479 (47.55)	
Diabetes, *n* (%)					< 0.0001
No	44,392 (86.88)	16,166 (92.94)	14,970 (86.73)	13,256 (77.92)	
Yes	9,769 (13.12)	1,828 (7.06)	3,058 (13.27)	4,883 (22.08)	
Asthma, *n* (%)					< 0.0001
No	46,748 (85.89)	15,913 (87.75)	15,707 (86.05)	15,128 (82.89)	
Yes	7,413 (14.11)	2,081 (12.25)	2,321 (13.95)	3,011 (17.11)	

RAR, red cell distribution width to albumin ratio; BMI, body mass index; RDW, red cell distribution width.

### Logit analysis

Table 2 summarized the analysis results from three models. Each model, whether crude, model 1, or model 2, pointed to a significant increase in asthma risk with higher RAR levels, reporting OR values of 1.48 (1.36–1.61, *P*_*trend*_ < 0.0001), 1.63 (1.49–1.78, *P*_*trend*_ < 0.0001), and 1.43 (1.30–1.58, *P*_*trend*_ < 0.0001). To put it another way, with the lowest RAR level group as the reference, the asthma risk in the high RAR group increased by 43%. Furthermore, Table 2 also illustrated the influence of dynamic changes in RAR on asthma. With every 0.5 unit increase in RAR, the risk of asthma escalated by 82% (1.55–2.12). Therefore, whether RAR was incorporated as a categorical or continuous variable, it significantly impacted asthma risk. Table 3 particularly showcased the multivariable logit regression results from model 2. The low RAR group serves as the reference, the moderate and high groups significantly elevated asthma risk, with respective ORs of 1.16 (1.06–1.27, *P*_*trend*_ = 0.002) and 1.43 (1.30–1.58, *P*_*trend*_ < 0.0001). Likewise, Supplementary Table 2 highlighted a significant correlation between elevated RAR and the increased occurrence of asthma.

**TABLE 2:** Relationships between RAR and asthma.

RAR	OR, 95% CI		
	**Crude model**	**Model 1**	**Model 2**
Low group	Ref	Ref	Ref
Moderate group	1.16 (1.06, 1.27)	1.24 (1.13, 1.35)	1.16 (1.06, 1.27)
High group	1.48 (1.36, 1.61)	1.63 (1.49, 1.78)	1.43 (1.30, 1.58)
Per 0.5 U increment	1.92 (1.69, 2.18)	2.17 (1.89, 2.50)	1.82 (1.55, 2.12)
*P* for trend	< 0.0001	< 0.0001	< 0.0001

RAR, red cell distribution width to albumin ratio; BMI, body mass index. Model 1: RAR, age, gender, and race. Model 2: RAR, age, gender, race, BMI, smoke status, hypertension, diabetes, eosinophil percentage, and globulin.

**TABLE 3:** Multivariate logit analysis of RAR and risk of asthma.

Characteristics	OR	95% CI	*P*
**RAR**			
Low group	Ref	Ref	
Moderate group	1.16	1.06, 1.27	0.002
High group	1.43	1.30, 1.58	< 0.0001
Age	0.98	0.98, 0.99	< 0.0001
**Gender**			
Male	Ref	Ref	
Female	1.47	1.37, 1.57	< 0.0001
**Race**			
White people	Ref	Ref	
Non-White people	0.85	0.78, 0.92	< 0.0001
BMI	1.02	1.02, 1.03	< 0.0001
**Smoke status**			
No/former	Ref	Ref	
Yes	1.17	1.07, 1.27	< 0.001
**Hypertension**			
No	Ref	Ref	
Yes	1.32	1.22, 1.42	< 0.0001
**Diabetes**			
No	Ref	Ref	
Yes	1.14	1.03, 1.26	0.01
Eosinophil percentage	1.12	1.11, 1.14	< 0.0001
Globulin	0.81	0.75, 0.89	< 0.0001

RAR, red cell distribution width to albumin ratio; BMI, body mass index.

### Non-linear relationships

**FIGURE 3:**
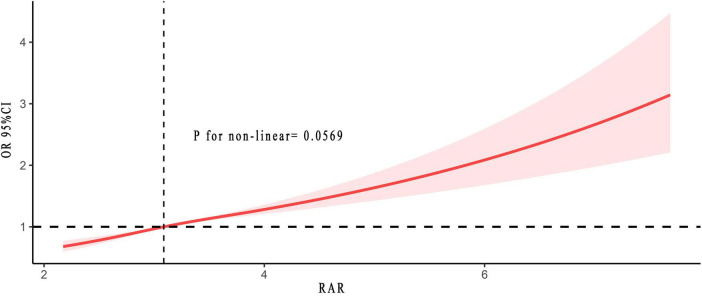
Non-linear relationship between RAR and asthma risk. The solid and red shadow represented the estimated values and their 95% CIs, respectively. RAR, red cell distribution width to albumin ratio.

Another significant finding of this study was the non-linear relationship. Figure 3 revealed no non-linear relationship between RAR and the risk of asthma (*p* for non-linear = 0.057). As RAR increases, the associated risk of asthma likewise escalated. They exhibited a positive correlation. The results in Supplementary Figure 1 further validated this finding.

### Sensitivity analysis

Table 4 consolidated the findings from stratified and interaction analyses. From Table 4, it could be seen that age, gender, race, smoking history, diabetes history, and eosinophil percentage showed no interaction with RAR, implying that regardless of whether the age was over or under 60, male or female, White people or Non-White people, with or without a history of smoking or diabetes, high or low eosinophil percentage, as RAR increased, so did the risk of asthma. Interactions might exist between BMI, hypertension, and globulin with RAR, however, it should be noted that regardless of whether BMI was above or below 25, with or without hypertension, and regardless of globulin levels being higher or lower than 3, the risk of asthma increased with increasing RAR. In conclusion, all subgroups in this study followed a consistent trend with the general population, indicating that an increase in RAR was a risk factor for asthma. The findings presented in Supplementary Table 3 provided additional confirmation of this result.

**TABLE 4:** Stratified analyses of the relationships between RAR and asthma risk.

Characteristics	RAR				
	**Low group**	**Moderate group**	**High group**	***P* for trend**	***P* for interaction**
Age					0.67
≤ 60	Ref	1.17 (1.06, 1.29)	1.47 (1.31, 1.64)	< 0.0001	
> 60	Ref	1.06 (0.88, 1.27)	1.38 (1.15, 1.65)	< 0.001	
Gender					0.05
Male	Ref	1.09 (0.97, 1.24)	1.37 (1.20, 1.57)	< 0.0001	
Female	Ref	1.20 (1.06, 1.36)	1.49 (1.33, 1.68)	< 0.0001	
Race					0.14
White people	Ref	1.16 (1.02, 1.30)	1.40 (1.24, 1.59)	< 0.0001	
Non-White people	Ref	1.12 (1.01, 1.25)	1.51 (1.36, 1.68)	< 0.0001	
BMI					0.004
< 25	Ref	1.04 (0.91, 1.19)	1.29 (1.10, 1.52)	0.01	
≥ 25	Ref	1.21 (1.08, 1.35)	1.49 (1.34, 1.67)	< 0.0001	
Smoke status					0.85
No/former	Ref	1.15 (1.04, 1.27)	1.45 (1.30, 1.61)	< 0.0001	
Yes	Ref	1.11 (0.94, 1.33)	1.42 (1.20, 1.68)	< 0.0001	
Hypertension					0.005
No	Ref	1.11 (1.00, 1.24)	1.36 (1.20, 1.55)	< 0.0001	
Yes	Ref	1.23 (1.08, 1.41)	1.59 (1.41, 1.81)	< 0.0001	
Diabetes					0.06
No	Ref	1.14 (1.04, 1.26)	1.41 (1.28, 1.55)	< 0.0001	
Yes	Ref	1.23 (0.96, 1.57)	1.68 (1.31, 2.14)	< 0.0001	
Eosinophil percentage					0.36
≤ 8	Ref	1.13 (1.03, 1.23)	1.43 (1.30, 1.57)	< 0.0001	
> 8	Ref	1.51 (0.98, 2.32)	1.72 (1.11, 2.67)	0.01	
Globulin					0.04
≤ 3	Ref	1.08 (0.97, 1.20)	1.37 (1.21, 1.54)	< 0.0001	
> 3	Ref	1.34 (1.14, 1.58)	1.62 (1.39, 1.89)	< 0.0001	

RAR, red cell distribution width to albumin ratio; BMI, body mass index.

## Discussion

This was the inaugural study investigating the link between RAR and asthma risk. The study revealed a direct correlation where higher RAR levels correspond to higher asthma risks. Asthma risk was 43% (1.30–1.58) higher in the group with elevated RAR compared to those with the lowest RAR. Each 0.5 unit increase in RAR triggered in an 82% (1.55–2.12) higher risk of asthma. Furthermore, for the first time, it was definitively shown that there was no non-linear correlation between RAR and asthma risk, meaning increased RAR consistently raised asthma risk without any turning point. Integrating subgroup and interaction analyses, all subgroups consistently demonstrate that higher RAR levels posed a risk factor for asthma, ensuring stable and reliable findings.

Asthma, a chronic inflammatory disease of the respiratory tract, manifested primarily through coughing, wheezing, breathlessness, and chest tightness. The symptoms of asthma arose from airway inflammation, triggering processes such as mucus production, airway wall remodeling, and heightened bronchial reactivity (25). The process involved activating a wide range of immune cells and releasing inflammatory factors, including eosinophils, neutrophils, lymphocytes, mast cells, and interleukins 15, 13, and 33 (25–27). This evidence underscored the critical importance of inflammation in the pathogenesis of asthma. Hence, identifying a stable, inexpensive, and comprehensive inflammatory biomarker was of paramount importance.

RAR represented a novel comprehensive inflammatory biomarker, which has considerable potential in predicting inflammatory conditions. Earlier research has linked RAR to a variety of inflammatory diseases, such as diabetes, stroke, sepsis, and chronic kidney disease (28–31). This research initially demonstrated that RAR significantly affected the risk of asthma, a chronic inflammatory respiratory condition, with increased RAR levels associated with heightened asthma risk [16% (1.06–1.27) and 43% (1.30–1.58) increased risk in the moderate and high groups, respectively, compared to the lowest group]. An increase of 0.5 unit in RAR almost doubled the asthma risk (OR: 1.82, 1.55–2.12). Furthermore, there was no non-linear relationship between RAR and asthma risk, implying that as RAR increased, asthma risk also grew. RAR acted as a predictive risk factor for asthma for primarily two reasons. On one hand, serum albumin has significant anti-inflammatory and antioxidant effects. Past studies indicated a negative relationship between albumin and the proportion of eosinophils, important inflammatory cells in asthma attacks (32). The excessive buildup of eosinophils in tissues was a major trigger for asthma, releasing leukotrienes that caused contraction of the bronchial smooth muscles, thus worsening asthma symptoms and advancing the inflammatory response (33). Furthermore, albumin also played an essential role in various inflammatory diseases, such as ST-elevation myocardial infarction, coronary artery disease, and diabetes (14, 34, 35). On the other hand, high RDW levels were linked to oxidative stress and inflammation (3, 36). Oxidative stress profoundly influenced the stability and survival of red blood cells (RBC), which accelerated the renewal of RBC, leading to increased RDW (37). Inflammatory cytokines inhibited the maturation of RBC in the bone marrow, releasing immature cells into the bloodstream, thus increasing the heterogeneity of RBC and elevating RDW levels (38, 39). Prospective, large-sample clinical studies were required in the future to further substantiate our results.

Our study identified several high-risk asthma groups that were more susceptible to changes in RAR, such as adult women, obese individuals, White people, smokers, and those with a history of hypertension or diabetes. These findings provided further validation of previous research outcomes (2, 40). Notably, the study indicated that with each year increase in age, the risk of asthma decreased by 2% (95% CI: 0.98–0.99). Potential reasons for this might include, firstly, that with aging, airway development and immune system maturation reduced the response to allergens. Additionally, asthma in adolescents and young adults was strongly associated with hormone levels, particularly in females. With increasing age, especially after women reach menopause, changed in hormone levels might influence airway allergic responses, thereby reducing asthma risk (41). Finally, with aging, individuals might distance themselves from allergens or harmful environments (air pollution and secondhand smoke) that were present in earlier life. A decrease in these environmental factors contributed to a reduced risk of asthma exacerbations.

Three aspects underscored the advantages of this study. Firstly, the study featured a large, representative data sample that was readily extractable and was dynamically updated every two years. As a result, the findings were highly reliable. Second, the study eliminated as many confounding factors as possible, based on various statistical methods (multivariate, subgroup, and interaction analyses). Finally, the components of RAR were readily accessible, affordable, and widely utilized clinically, encouraging their extensive application in clinical environments.

One should be aware that this study has certain limitations as well. One limitation, due to the observational nature and cross-sectional setting of this study, was that the causal relationship between RAR and asthma risk could not be clearly established. We recommend that future research be prospective to clarify their causal relationship. Conversely, it was impossible to entirely eliminate confounding factors. Hence, we have utilized various methods to minimize them as much as possible. Finally, asthma diagnosis was determined by self-report, and the data were from a single US cohort, which might affect the results. Future multicenter, prospective research was necessary to verify these findings.

## Conclusion

It should be highlighted that this was the first article to investigate the relationship between RAR and asthma risk, revealing a linear positive correlation between them. As RAR levels rose, irrespective of whether RAR was included as a categorical or continuous variable in analyses, the risk of asthma markedly increased. Additional analysis confirmed that no non-linear relationship existed between the two. This implied that for populations at high risk for asthma, clinicians should focus more on their RAR levels to predict the likelihood of asthma onset more effectively. For instance, if serum albumin level was found to be low, adequate supplementation with serum albumin should be provided. In cases of elevated RDW, it was important to identify the underlying cause and implement targeted treatment to return RDW to a normal range, while maintaining RAR within a relatively low range. Considering the numerous benefits of RAR, it held promising prospects for clinical application.

## Data Availability

The raw data supporting the conclusions of this article will be made available by the authors, without undue reservation.
