# Corrigendum: Red cell distribution width to albumin ratio is associated with asthma risk: a population-based study

**DOI:** 10.3389/fmed.2025.1571811

**Published:** 2025-02-18

**Authors:** Jinzhen Ding, Yixiang Zhang, Xiaoyang Chen

**Affiliations:** ^1^Department of Pulmonary and Critical Care Medicine, The Second Affiliated Hospital of Fujian Medical University, Quanzhou, Fujian, China; ^2^Respirology Medicine Centre of Fujian Province, The Second Affiliated Hospital of Fujian Medical University, Quanzhou, China

**Keywords:** red cell distribution width to albumin ratio, asthma, NHANES, risk, inflammation

In the published article, there were errors in the affiliations. For affiliation 1, instead of “Department of Pulmonary and Critical Care Medicine, The Second Affiliated Hospital of Fujian, Quanzhou, Fujian, China,” it should be “Department of Pulmonary and Critical Care Medicine, The Second Affiliated Hospital of Fujian Medical University, Quanzhou, Fujian, China.” For affiliation 2, instead of “Respirology Medicine Centre of Fujian Province, Medical University, Quanzhou, China,” it should be “Respirology Medicine Centre of Fujian Province, The Second Affiliated Hospital of Fujian Medical University, Quanzhou, China.”

The authors apologize for this error and state that this does not change the scientific conclusions of the article in any way. The original article has been updated.

