# Supplementary material for: Red cell distribution width to albumin ratio is associated with asthma risk: a population-based study
Source: Front Med (Lausanne). 2024 Dec 11;11:1493463. doi: 10.3389/fmed.2024.1493463 (PMC11668568; doi:10.3389/fmed.2024.1493463)
Supplement: Supplementary file 1 [file Table_1.DOC]

**Supplementary files**

**Supplementary Table S1. Baseline characteristics of patients with asthma**

**Supplementary Table S2. Relationships bewteen RAR and asthma**

**Supplementary Table S3. Multivariate logit analysis of RAR and risk of asthma**

**Supplementary Figure S1. Non-linear relationship between RAR and asthma risk.**

**Supplementary Table S1. Baseline characteristics of patients with asthma**

| **Characteristics** | **Total** | **Without asthma** | **With asthma** | ***P*** |
| --- | --- | --- | --- | --- |
| Participants, n | 26707 | 23449 | 3258 |  |
| RAR, mean (SD) | 2.97(0.00) | 2.96(0.00) | 3.01(0.01) | < 0.0001 |
| RAR, n (%) |  |  |  | < 0.0001 |
| Low group | 8897(40.30) | 7919(40.88) | 978(36.46) |  |
| Moderate group | 8912(32.95) | 7901(33.09) | 1011(31.98) |  |
| High group | 8898(26.75) | 7629(26.03) | 1269(31.56) |  |
| Age, year (SD) | 46.68(0.21) | 46.97(0.22) | 44.72(0.34) | < 0.0001 |
| Gender, n (%) |  |  |  | < 0.0001 |
| Male | 13364(48.96) | 11976(50.02) | 1388(41.85) |  |
| Female | 13343(51.04) | 11473(49.98) | 1870(58.15) |  |
| Race, n (%) |  |  |  | 0.004 |
| White people | 13339(71.59) | 11555(71.26) | 1784(73.78) |  |
| Non-White people | 13368(28.41) | 11894(28.74) | 1474(26.22) |  |
| Educational status |  |  |  | < 0.001 |
| ≥College or above | 3745( 6.67) | 3441(6.96) | 304(4.67) |  |
| High school or equivalent | 12236(55.43) | 10590(55.02) | 1646(58.14) |  |
| <High school | 10726(37.91) | 9418(38.01) | 1308(37.19) |  |
| BMI, Kg/m2 (SD) | 28.35(0.07) | 28.18(0.08) | 29.47(0.18) | < 0.0001 |
| Albumin, g/dL (SD) | 4.31(0.01) | 4.31(0.01) | 4.26(0.01) | < 0.0001 |
| RDW, % (SD) | 12.69(0.01) | 12.68(0.01) | 12.75(0.03) | 0.01 |
| Eosinophil percentage, % (SD) | 2.85(0.01) | 2.78(0.01) | 3.29(0.05) | < 0.0001 |
| Globulin, g/dL (SD) | 2.92(0.01) | 2.92(0.01) | 2.91(0.01) | 0.28 |
| CRP, mg/dL (SD) | 0.40(0.01) | 0.39(0.01) | 0.48(0.02) | < 0.0001 |
| NLR, mean (SD) | 2.20(0.01) | 2.20(0.01) | 2.20(0.03) | 0.98 |
| Smoke status, n (%) |  |  |  | 0.001 |
| No/former | 20720(76.41) | 18310(76.84) | 2410(73.56) |  |
| Yes | 5987(23.59) | 5139(23.16) | 848(26.44) |  |
| Hypertension, n (%) |  |  |  | 0.02 |
| No | 15600(64.54) | 13810(64.91) | 1790(62.08) |  |
| Yes | 11107(35.46) | 9639(35.09) | 1468(37.92) |  |
| Diabetes, n (%) |  |  |  | 0.02 |
| No | 22496(88.85) | 19826(89.10) | 2670(87.23) |  |
| Yes | 4211(11.15) | 3623(10.90) | 588(12.77) |  |
| Stroke, n (%) |  |  |  | < 0.0001 |
| No | 25715(97.31) | 22611(97.50) | 3104(96.05) |  |
| Yes | 992( 2.69) | 838(2.50) | 154(3.95) |  |
| CVD |  |  |  | < 0.0001 |
| No | 23652(91.42) | 20894(91.91) | 2758(88.13) |  |
| Yes | 3055( 8.58) | 2555( 8.09) | 500(11.87) |  |
| Cancer |  |  |  | < 0.001 |
| No | 24278(91.48) | 21370(91.75) | 2908(89.65) |  |
| Yes | 2429( 8.52) | 2079( 8.25) | 350(10.35) |  |

Abbreviations: RAR, red cell distribution width to albumin ratio; BMI, body mass index; RDW, red cell distribution width; CRP, C-reactive protein; NLR, neutrophil to lymphocyte ratio; CVD, cardiovascular disease.

**Supplementary Table S2. Relationships bewteen RAR and asthma**

| **RAR** | **OR, 95%CI** | | |
| --- | --- | --- | --- |
|  | **Crude Model** | **Model 1** | **Model 2** |
| Low group | ref | ref | ref |
| Moderate group | 1.08(0.97,1.22) | 1.14(1.02,1.29) | 1.06(0.94,1.19) |
| High group | 1.36(1.21,1.52) | 1.48(1.32,1.66) | 1.19(1.05,1.34) |
| Per 0.5 U increment | 1.78(1.47,2.14) | 2.06(1.72,2.46) | 1.40(1.11,1.75) |
| ***P* for trend** | <0.0001 | <0.0001 | 0.01 |

Abbreviations: RAR, red cell distribution width to albumin ratio; BMI, body mass index.

Model 1: RAR, age, gender, race, and education status.

Model 2: RAR, age, gender, race, education status, BMI, smoke status, hypertension, diabetes, stroke, cardiovascular disease, cancer, eosinophil percentage, globulin, C-reactive protein, and neutrophil to lymphocyte ratio.

**Supplementary Table S3. Multivariate logit analysis of RAR and risk of asthma**

| **Characteristics** | **OR** | **95% CI** | ***P*** |
| --- | --- | --- | --- |
| RAR |  |  |  |
| Low group | ref | ref |  |
| Moderate group | 1.06 | 0.94,1.19 | 0.37 |
| High group | 1.19 | 1.05,1.34 | 0.01 |
| Age | 0.98 | 0.98,0.98 | <0.0001 |
| Gender |  |  |  |
| Male | ref | ref |  |
| Female | 1.51 | 1.38,1.64 | <0.0001 |
| Race |  |  |  |
| White people | ref | ref |  |
| Non-White people | 0.87 | 0.79,0.96 | 0.01 |
| Educational status |  |  |  |
| ≥College  or above | ref | ref |  |
| High school  or equivalent | 1.50 | 1.26,1.77 | <0.0001 |
| <High school | 1.32 | 1.10,1.59 | 0.003 |
| BMI | 1.02 | 1.02,1.03 | <0.0001 |
| Smoke status |  |  |  |
| No/former | ref | ref |  |
| Yes | 1.17 | 1.05,1.31 | 0.01 |
| Hypertension |  |  |  |
| No | ref | ref |  |
| Yes | 1.20 | 1.09,1.32 | <0.001 |
| Diabetes |  |  |  |
| No | ref | ref |  |
| Yes | 1.16 | 0.98,1.37 | 0.09 |
| Stroke |  |  |  |
| No | ref | ref |  |
| Yes | 1.05 | 0.81,1.35 | 0.73 |
| CVD |  |  |  |
| No | ref | ref |  |
| Yes | 1.78 | 1.47,2.15 | <0.0001 |
| Cancer |  |  |  |
| No | ref | ref |  |
| Yes | 1.41 | 1.22,1.63 | <0.0001 |
| Eosinophil percentage | 1.12 | 1.11,1.14 | <0.0001 |
| Globulin | 0.81 | 0.75,0.89 | <0.0001 |
| CRP | 1.06 | 1.02,1.11 | 0.01 |
| NLR | 1.02 | 0.97,1.07 | 0.4 |

Abbreviations: OR, odds ratio; CI, Confidence interval; RAR, red cell distribution width to albumin ratio; BMI, body mass index; CVD, cardiovascular disease; CRP, C-reactive protein; NLR, neutrophil to lymphocyte ratio.


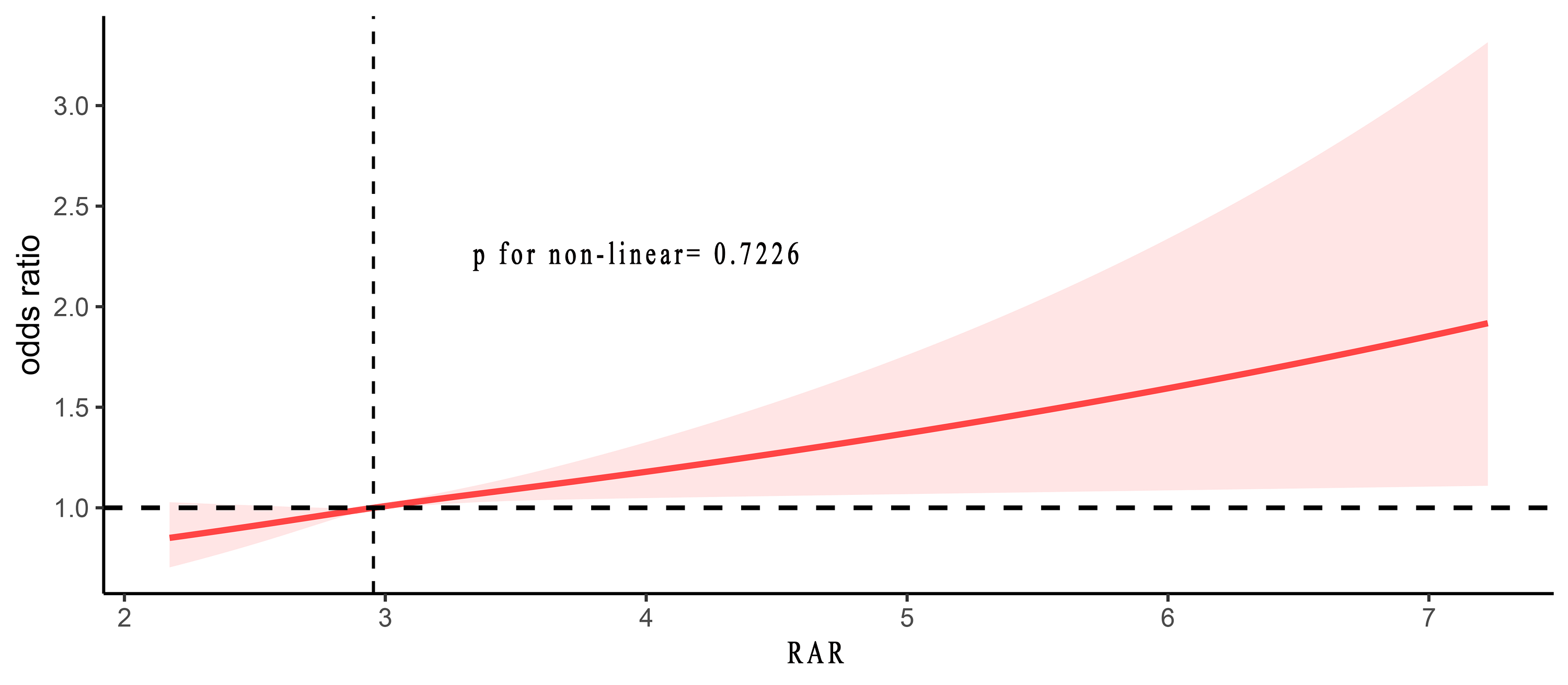


**Supplementary Figure S1. Non-linear relationship between RAR and asthma risk.** The solid and red shadow represented the estimated values and their 95% CIs, respectively. RAR, red cell distribution width to albumin ratio.
